# Supplementary material for: French Survey on Pain Perception and Management in Patients with Locked-In Syndrome
Source: Diagnostics (Basel). 2022 Mar 21;12(3):769. doi: 10.3390/diagnostics12030769 (PMC8947195; doi:10.3390/diagnostics12030769)
Supplement: Supplementary file 1 [file diagnostics-12-00769-s001.zip › Supplementary/Table S1.pdf]

Table S1. Questions included in the survey (VAS = Visual Analogue Scale, SD = standard deviation).

Items and responses	Total of respondents	Mean VAS score ± SD	Frequency (%)
**1. Have you felt pain or physical discomfort these last two weeks?**	**51**		
Yes	25		49
No	26		51
**2. If you feel pain, on which place(s) of your body is it? (several choices possible)**	**25**		
Lower limb	21		84
Upper limb	6		24
Abdominals	4		16
Back	5		20
Head	7		28
Diffuse	2		8
**3. Indicate on a scale ranging from 0 to 10 the intensity of the MOST INTENSE PAIN felt these last two weeks (0 = no pain, 10 = most intense pain)**	**25**	6.16 ± 2.44	
**4. Indicate on a scale ranging from 0 to 10 the intensity of the LESS INTENSE PAIN felt these last two weeks (0 = no pain, 10 = most intense pain)**	**25**	1.88 ± 2.55	
**5. Indicate on a scale ranging from 0 to 10 the intensity of the MEAN PAIN felt these last two weeks (0 = no pain, 10 = most intense pain)**	**25**	4.20 ± 1.98	
**6. Does the pain have one or more of the following characteristics? (several choices possible)**	**25**		
0 = no features	10		40
1 = burn	6		24
2 = painful cold sensation	4		16
3 = electrical shocks	10		40
**7. Do you experience other symptom(s) in the painful area? (several choices possible)**	**25**		
None	9		36
Tingling	5		20
Pinprick	6		24
Numbness	6		24
Itches	3		12
Feeling of vice	12		48
**8. Do you feel a decrease in touch sensitivity in areas where pain is present?**	**25**		
Decreased sensitivity during sting	20		80
Decreased sensitivity during simple touch	3		12
No decrease in sensitivity	2		8
**9. Is pain caused or increased by friction?**	**25**		
Yes	8		32
No	17		68
**10. Indicate since when you feel these pains:**	**25**		
1-15 days	0		0
15 days - 1month	0		0
1 -3 months	2		8
3 -6 months	6		24
6 months -1 year	5		20
> 1 year	12		48
**11. Before the locked-in syndrome, did you already have these pains?**	**25**		
Yes	2		8

No	21		84
I do not know	2		8
**12. If you already had pain before the locked-in syndrome, indicate on a scale ranging from 0 to 10 the intensity of this pain (0 = no pain, 10 = most intense pain)**	**25**	2.5 ± 2.08	
**13. If you had this pain before, how did your pain progress following the locked-in syndrome?**	**4**		
Aggravation of pain	0		0
Decrease of pain	2		50
Same pain	1		25
I do not know	0		0
**14. The pain you are currently experiencing is:**	**25**		
Punctual	21		84
Continue	4		16
**If punctual, do you feel it:**	**21**		
More than once a day	4		19
Once a day	2		9
Less than once a day	3		14.
I do not know	12		57
**If punctual, do you feel it more:**	**21**		
The morning	5		23
The afternoon	5		23
The evening	6		28
I do not know	5		23
**15. When you are in pain, how do you express pain? (several choices possible)**	**25**		
Winces	6		24
Cries	11		44
Look/blinking	4		16
Vocalizations	7		28
Communication code	7		28
No expression of pain	13		52
Other	2		8
**16. When you feel pain, do you feel that certain elements increase the pain? (several choices possible)**	**25**		
Mood/emotions	**1**		4
Temperature	4		16
Supine	3		12
Sitting	7		28
Care (nurses, physiotherapy, ...)	8		32
Touching	10		40
Tiredness	10		40
Physical exercises	8		32
Equipment (specific cushion, …)	2		8
None	2		8
Other	3		12
**17. When you feel pain, do you feel that certain elements decrease the pain? (several choices possible)**	**25**		
Mood/emotions	0		0
Temperature	1		4
Supine	4		16
Sitting	6		24
Care (nurses, physiotherapy, ...)	2		8

Touching	10		40
Tiredness	5		20
Physical exercises	6		24
Equipment (specific cushion, …)	3		12
None	3		12
Other	9		36
**18. When you have pain, do you feel that pain affects your mental abilities? If yes, specify (several choices possible)**	**25**		
Decreased concentration/attention	4		16
Increased mood swings	13		52
Decreased memory capacity	4		16
Depression	6		24
Tiredness	14		56
None	9		36
**19. Does the pain disrupt your sleep?**	**25**		
Yes	10		40
No	13		52
I do not know	2		8
**20. If yes, indicate on a scale ranging from 0 to 10 how much the pain disturbs your sleep (0 = no influence, 1 = strong influence)**	**25**	6.42 ± 3.23	
**21. When you are in pain, do you feel that pain affects your emotions?**	**25**		
Always	3		12
Sometimes	4		16
Rarely	14		56
Never	4		16
**22. If yes, indicate on a scale ranging from 0 to 10 how pain affects your emotions (0 = no influence, 1 = strong influence)**	**25**	4.68 ± 2.92	
**23. Do you take any pharmacological treatment to ease your pain?**	**25**		
Yes	15		60
No	10		40
**24. If yes, how often do you take this treatment?**	**15**		
Several times a day	5		33
Once a day	8		53
Occasionally, when the pain is too strong	2		13
**25. If yes, indicate on a scale ranging from 0 to 10 how effective this (these) treatment(s) is (are) (0 = no influence, 1 = strong influence)**	**25**	6 ± 2.93	
**26. To relieve your pain, have you ever tried non-pharmacological treatments (example: meditation, hypnosis, etc.)?**	**25**		
Yes	3		12
No	21		84
I do not know	1		4
**27. If yes, indicate on a scale ranging from 0 to 10 how effective this (these) treatment(s) is (are) (0 = no influence, 1 = strong influence)**	**25**	2 ± 1.73	
**28. Do you want to test a new pharmacological or non-pharmacological treatment? (several choices possible)**	**25**		
Yes, I would be ready to test a new pharmacological treatment	12		48
Yes, I would be ready to test a new non-pharmacological treatment	6		24
No, I wouldn’t be ready to test a new pharmacological treatment	7		28
No, I wouldn’t be ready to test a new non-pharmacological treatment	14		56
