# Supplementary material for: French Survey on Pain Perception and Management in Patients with Locked-In Syndrome
Source: Diagnostics (Basel). 2022 Mar 21;12(3):769. doi: 10.3390/diagnostics12030769 (PMC8947195; doi:10.3390/diagnostics12030769)
Supplement: Supplementary file 1 [file diagnostics-12-00769-s001.zip › Supplementary/Table S2.pdf]

Table S2. Bivariate analysis of the relationship between the presence or absence of pain and demographic information of the patients included in the study.

Variable	Overall, N = 51	Presence of pain		p-value
		**No**, N = 26	**Yes**, N = 25	
**Sex, n (%)**				0,31[1]
Female	18 (50%)	8 (40%)	10 (62%)	
Male	18 (50%)	12 (60%)	6 (38%)	
Unknown	15	6	9	
**Etiology, n (%)**				0,57[2]
Infection	2 (4%)	2 (8%)	0 (0%)	
Other	4 (8%)	2 (8%)	2 (8%)	
Stroke	41 (80%)	20 (77%)	21 (84%)	
Traumatic brain injury	4 (7%)	2 (8%)	2 (8%)	
**Tracheotomy, n (%)**				0,21[2]
No	25 (49%)	15 (29%)	10 (20%)	
Yes	26 (51%)	11 (22%)	15 (29%)	
**Gastrostomy, n (%)**				0,17[1]
No	17 (33%)	11 (22%)	6 (12%)	
Yes	34 (67%)	15 (29%)	19 (37%)	
**Time since injury, Median (IQR)**	9 (6 – 18)	10 (6 – 16)	6 (3 – 18)	0,26[3]

[1] Pearson's Chi-squared test; [2]Fisher's exact test; [3]Wilcoxon rank sum test
