# Supplementary material for: French Survey on Pain Perception and Management in Patients with Locked-In Syndrome
Source: Diagnostics (Basel). 2022 Mar 21;12(3):769. doi: 10.3390/diagnostics12030769 (PMC8947195; doi:10.3390/diagnostics12030769)
Supplement: Supplementary file 1 [file diagnostics-12-00769-s001.zip › Supplementary/Table S4.pdf]

Table S4. Bivariate analysis of the relationship between the use of pain treatments and the presence of sleep disturbance or cognitive disabilities, or pain intensity.

Variable, n (%)	Overall, N =25	Use of pharmacological treatment		p-value[1]	Overall, N =25	Use of non-pharmacological treatment			p-value[1]
		**No**, N = 10	**Yes**, N = 15			**No**, N = 21	**Yes**, N = 3	**Do not know**, N = 1	
**Influence on sleep,**				0.33					0.4
No	13 (52%)	5 (20%)	8 (32%)		13 (52%)	10 (40%)	3 (12%)	0 (0%)	
Yes	10 (40%)	3 (12%)	7 (28%)		10 (40%)	9 (36%)	0 (0%)	1 (4%)	
Do not know	2 (8%)	2 (8%)	0 (0%)		2 (8%)	2 (8%)	0 (0%)	0 (0%)	
**Decrease in concentration/attention**				0.1					0.76
No	11 (44%)	2 (8%)	9 (36%)		11 (44%)	9 (36%)	1 (4%)	1 (4%)	
Yes	14 (56%)	8 (32%)	6 (24%)		14 (56%)	12 (48%)	2 (8%)	0 (0%)	
**Increase in mood swings**				0.69					0.70
No	16 (64%)	7 (28%)	9 (36%)		16 (64%)	14 (56%)	2 (8%)	0 (0%)	
Yes	9 (36%)	3 (12%)	6 (24%)		9 (36%)	7 (28%)	1 (4%)	1 (4%)	
**Decreased memory capacity**				0.18					0.66
No	19 (76%)	6 (24%)	13 (52%)		19 (76%)	15 (60%)	3 (12%)	1 (4%)	
Yes	6 (24%)	4 (16%)	2 (8%)		6 (24%)	6 (24%)	0 (0%)	0 (0%)	
**Tiredness**				0.43					0.39
No	12 (48%)	6 (24%)	6 (24%)		12 (48%)	9 (36%)	2 (8%)	1 (4%)	
Yes	13 (52%)	4 (16%)	9 (36%)		13 (52%)	12 (48%)	1 (4%)	0 (0%)	
**Depression**				0.63					0.21
No	21 (84%)	9 (36%)	12 (48%)		21 (84%)	18 (72%)	3 (12%)	0 (0%)	
Yes	4 (16%)	1 (4%)	3 (12%)		4 (16%)	3 (12%)	0 (0%)	1 (4%)	
**None**				0.63					0.53
No	21 (84%)	9 (36%)	12 (48%)		21 (84%)	18 (72%)	2 (8%)	1 (4%)	
Yes	4 (16%)	1 (4%)	3 (12%)		4 (16%)	3 (12%)	1 (4%)	0 (0%)	
**Pain intensity**				> 0.99					0.17
Greater or equal to 5	18 (72%)	7 (28%)	11 (44%)		18 (72%)	15 (60%)	3 (12%)	0 (0%)	
Lower than 5	7 (28%)	3 (12%)	4 (16%)		7 (28%)	6 (24%)	0 (0%)	1 (4%)	

[1]Fisher's exact test
